# Tobacco, but Not Nicotine and Flavor-Less Electronic Cigarettes, Induces ACE2 and Immune Dysregulation

**DOI:** 10.3390/ijms21155513

**Published:** 2020-07-31

**Authors:** Abby C. Lee, Jaideep Chakladar, Wei Tse Li, Chengyu Chen, Eric Y. Chang, Jessica Wang-Rodriguez, Weg M. Ongkeko

**Affiliations:** 1 Division of Otolaryngology-Head and Neck Surgery, Department of Surgery, University of California San Diego, La Jolla, CA 92093, USA; acl008@ucsd.edu (A.C.L.); jchaklad@ucsd.edu (J.C.); wtl008@ucsd.edu (W.T.L.); chc401@ucsd.edu (C.C.); 2 Research Service, VA San Diego Healthcare System San Diego, La Jolla, CA 92161, USA; 3 Department of Radiology, Radiology Service, VA San Diego Healthcare System San Diego, University of California San Diego, La Jolla, CA 92093, USA; Eric.Chang2@va.gov; 4 Department of Pathology, Pathology Service, VA San Diego Healthcare System San Diego, University of California San Diego, La Jolla, CA 92093, USA; Jessica.Wang-Rodriguez@va.gov

**Keywords:** electronic cigarettes, vaping, COVID-19, SARS-CoV-2, ACE2, tobacco, inflammation, cytokines, immune response

## Abstract

The COVID-19 pandemic caused by the SARS-CoV-2 virus, overlaps with the ongoing epidemics of cigarette smoking and electronic cigarette (e-cig) vaping. However, there is scarce data relating COVID-19 risks and outcome with cigarette or e-cig use. In this study, we mined three independent RNA expression datasets from smokers and vapers to understand the potential relationship between vaping/smoking and the dysregulation of key genes and pathways related to COVID-19. We found that smoking, but not vaping, upregulates ACE2, the cellular receptor that SARS-CoV-2 requires for infection. Both smoking and use of nicotine and flavor-containing e-cigs led to upregulation of pro-inflammatory cytokines and inflammasome-related genes. Specifically, chemokines including CCL20 and CXCL8 are upregulated in smokers, and CCL5 and CCR1 are upregulated in flavor/nicotine-containing e-cig users. We also found genes implicated in inflammasomes, such as CXCL1, CXCL2, NOD2, and ASC, to be upregulated in smokers and these e-cig users. Vaping flavor and nicotine-less e-cigs, however, did not lead to significant cytokine dysregulation and inflammasome activation. Release of inflammasome products, such as IL-1B, and cytokine storms are hallmarks of COVID-19 infection, especially in severe cases. Therefore, our findings demonstrated that smoking or vaping may critically exacerbate COVID-19-related inflammation or increase susceptibility to COVID-19.

## 1. Introduction

The current COVID-19 pandemic, caused by the severe acute respiratory syndrome coronavirus 2 (SARS-CoV-2), has killed over 130,000 Americans in less than 4 months. The majority are over 65 years old, but many younger patients who have underlying medical conditions required admission to the intensive care unit. Current data indicate that patients who have cardiovascular and chronic respiratory conditions, including those caused by tobacco use, are at higher risk of developing severe COVID-19 symptoms and have significantly increased fatality [1]. There are increasing numbers of reports that smokers have worse clinical outcomes when infected with SARS-CoV-2 [2]. Information from China shows that people who have cardiovascular and respiratory conditions caused by tobacco use are at higher risk of developing severe COVID-19 symptoms [2,3]. Furthermore, tobacco use is the most important risk-factor for chronic obstructive pulmonary disease (COPD) [4]. Finally, aside from lung damage, it is believed that smokers may be more vulnerable to SARS-CoV-2 because of an altered immune response [5]. However, many studies reported otherwise. In a preprint, Farsalinos et al. concluded that smoking is not a risk factor for hospitalization due to COVID-19 [6]. One meta-analysis reported no association between smoking and severity of COVID-19 [7]. A French study even found that nicotine may protect against COVID-19 infection, prompting interests from the French Health Minister in studying whether nicotine patches could be used for COVID-19 treatment and prevention [8,9]. Unfortunately, all reports regarding tobacco and COVID-19 are from epidemiological data and only a few studies reported data. The lack of well-controlled laboratory experiments renders it extremely difficult to determine whether tobacco truly affects COVID-19 outcome and the mechanistic link between them. Furthermore, no peer-reviewed study has reported definitive data demonstrating or rejecting the hypothesis that COVID-19 incidence can be increased by tobacco smoking.

Electronic cigarettes (e-cigs) have dramatically increased in popularity in recent years, especially among the youth [10], so considerable attention has been directed to any potential link between e-cig and COVID-19 in recent months. E-cigs have been shown to induce inflammation of human airways and may also increase susceptibility to pneumonia by increasing pneumococcal adherence to airway cells [11,12]. Notable damage to the lung epithelium could also be done by e-cigs, resulting in increased airway hyperactivity and mucine production [12]. The detrimental effects of e-cigs thus fueled speculation that e-cigs may increase COVID-19 severity or incidence. However, virtually no study, both on the basic science level and epidemiological level, has linked COVID-19 to e-cigs. Complicating research on e-cigs’ effects on COVID-19 is the fact that e-cigs can be vaped with or without nicotine and with or without flavorings. Flavors and nicotine in e-cigs are frequently associated with more inflammation, epithelial barrier dysregulation, oxidative stress, and DNA damage than e-cigs with only basic components [12,13].

Another possible mechanism linking tobacco smoking to COVID-19 is its propensity to increase lung inflammation. COVID-19 has a strong immunological component, and poor outcomes have recently been associated with cytokine storms and a hyperinflammatory immune system [14,15]. Tobacco is known to induce extensive immune dysregulation in smokers, and its influence has been found to lead to airway inflammation [16]. Although cigarette smoke has been associated with increased susceptibility to COVID-19 [17], the effect of tobacco on the immune response to SARS-CoV-2 has not been studied. E-cigs are also known to induce significant inflammation in the lungs, but the dysregulated immune landscape is different from that of tobacco [18,19]. Therefore, it is plausible that inflammation induced by tobacco or e-cigs could exacerbate inflammation caused by SARS-CoV-2 infection, potentially triggering a lethal cytokine storm.

In this study, we directly investigated the two main hypotheses that might link COVID-19 to e-cig vaping and tobacco smoking, ACE2 upregulation and inflammation, by reanalysis of three independent datasets of gene expression from e-cig vapers and tobacco smokers. We examined whether e-cigs or tobacco upregulate ACE2, dysregulate immune cell population levels, affect cytokine levels, and regulate the activation of inflammasomes. Inflammasomes are critical mediators of inflammation, and their activation would lead to the certain release of cytokines [20]. Since severe COVID-19 is marked by cytokine storms, we investigated both inflammasome activation and the release of cytokines. Given that nicotine or flavorings present in the e-cig mixture are implicated in promoting lung damage, we examined one e-cig dataset where all participants only vaped e-cigs with neither nicotine nor flavors and another e-cig dataset where participants only vaped e-cigs with nicotine and were free to choose different flavors.

## 2. Results

### 2.1. Correlation between Smoking/Vaping and ACE2 Expression in Bronchial Epithelial Cells

To observe the relationship between smoking e-cigarettes and ACE2 expression, we used the following datasets: GSE138326 and GSE112073 [21,22]. The GSE138326 dataset, from Song et al., details gene expression in the bronchial epithelial cells of patients who smoked flavor-less and nicotine-less e-cigs vs. those who did not. The GSE112073 dataset, from Corbett et al., details gene expression in bronchial cells of patients who smoked nicotine-containing e-cigs of any flavor vs. those who smoked no e-cigs. All patients in this dataset were former smokers. The Kruskal–Wallis analysis test was applied to determine differential expression of ACE2 between e-cig users and non-e-cig users for both of these datasets (*p* < 0.05). Both lung and bronchial epithelial samples, in particular bronchial transient secretory cells, have been found to express significant amounts of ACE2 [23]. In addition, whole transcriptome RNA sequencing data for normal bronchus and lung tissue samples of 49 lung squamous cell carcinoma (LUSC) patients from The Cancer Genome Atlas (TCGA) were downloaded. These patients are either current or former smokers. Former smokers were categorized into those who had quit less than 15 years from the date of sample collection and those who had quit more than 15 years from the date of sample collection. Differential expression analysis was conducted to compare gene expression in current smokers and former smokers (Kruskal–Wallis, *p* < 0.05). 

**Figure 1:**
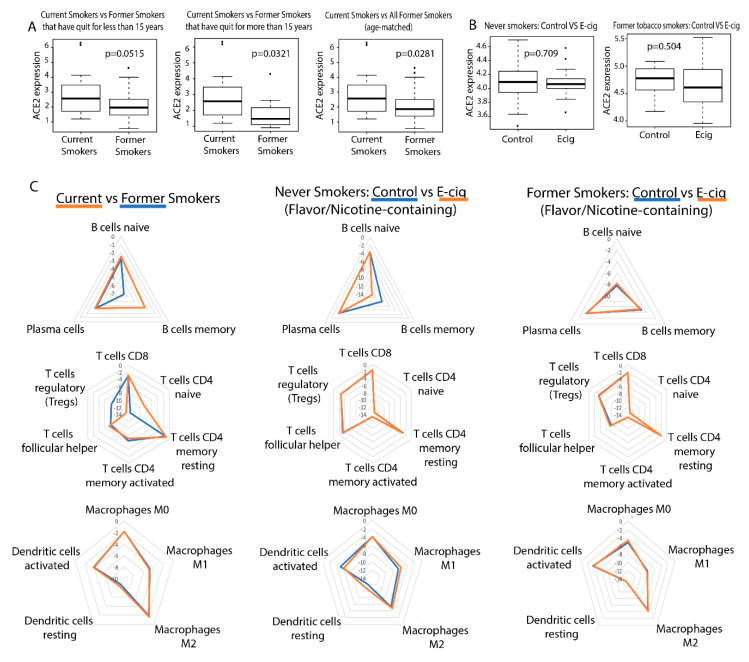
ACE2 and general immune dysregulation analysis in tobacco and electronic cigarette (e-cig) users. (**A**) Boxplots of ACE2 expression in current and former smokers, including those that have quit for less than and more than 15 years. (**B**) Boxplots of ACE2 expression in control and e-cigarette users, including patients who have never smoked tobacco and those who are former tobacco smokers. (**C**) Radar plots comparing immune cell infiltration across smokers, e-cigarette users vaping nicotine-less, flavor-less e-cigs, and vapers who used e-cigs containing nicotine and flavors. Greater immune cell infiltration is indicated by a value closer to zero and farther away from the center of the radar plot.

We found that smoking tobacco is associated with increased ACE2 expression in current smokers (*p* = 0.0281). In addition, the difference in ACE2 expression between current smokers and former smokers who had quit for more than 15 years is more significant than that of current smokers and former smokers who had quit for less than 15 years (Figure 1A). This is not surprising as we expect that a longer duration since quitting smoking would be associated with a greater difference in ACE2 expression between current and former smokers. On the other hand, smoking e-cigarettes does not lead to increased ACE2 expression in either study (Figure 1B). These results suggest that tobacco smokers may be more vulnerable to SARS-CoV-2 infection than e-cigarette smokers if infection susceptibility is based upon ACE2 abundance. We also found that TMPRSS2 expression is not dysregulated by e-cigs in either cohort but is dysregulated by smoking, as reported in our previous study [24].

### 2.2. Correlation between Smoking/Vaping Status and Immune Infiltration

Using the software tool CIBERSORTx [25], we deconvoluted the infiltrating lymphocyte population from bulk-tissue gene expression data (*p* < 0.05). We also found that smoking tobacco is associated with increased immune cell infiltration (Figure 1C). Current tobacco smokers experienced an upregulation of memory B-cell and naive CD4 T-cell expression and downregulation of regulatory T-cell (Treg) expression, compared to former smokers (Figure 1C). Elevated levels of CD4 T-cells in current tobacco smokers could lead to higher levels of effector cytokines that contribute to lung inflammation, making tobacco smokers more susceptible to a more aggressive immune response and a possible cytokine storm. In fact, it was reported that CD4+ T-cells contribute effector inflammation cytokines after acute influenza infections in mice, even after no virus is detectable in blood [26]. Meanwhile, the downregulation of Tregs may deprive the immune system of inhibitory signals that can prevent cytokine storms from occurring [27]. Memory B cells upregulation is not known to be involved in a cytokine storm, but their expansion may be indicative of past infections and increased susceptibility to infection in smokers. Our data is consistent with existing literature, which reports that current smokers have higher percentages of memory B cells than former smokers [28]. On the other hand, e-cig use is not significantly associated with increased immune infiltration in either datasets (Figure 1C).

### 2.3. Correlation between Smoking/Vaping Status and Cytokine Level Dysregulation

**Figure 2:**
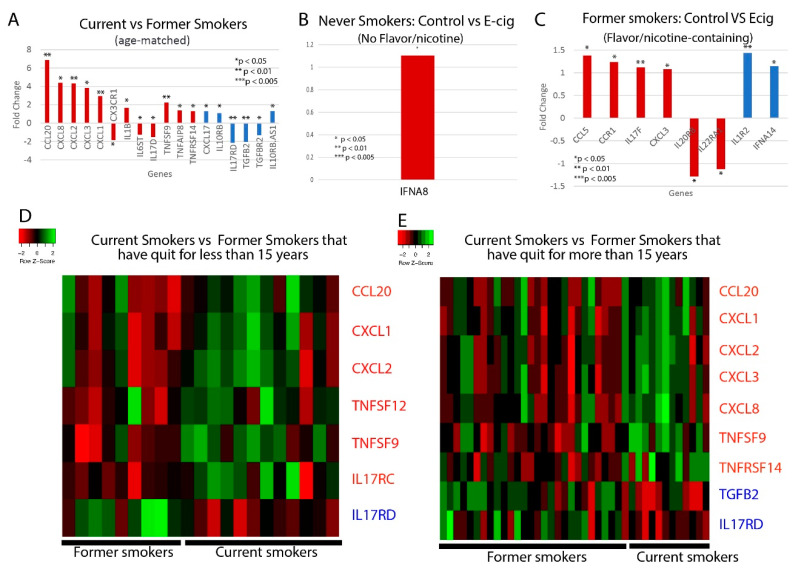
Cytokine-related gene expression. Bar plots of the fold changed of significantly dysregulated cytokine genes in (**A**) current vs. former smokers; and control vs. e-cig users that have used e-cigs with (**B**) no flavors or nicotine or (**C**) with nicotine and/or flavors. Heat maps illustrating differences in cytokine gene expression between current smoker and former smokers that have either quit for (**D**) less than 15 years or (**E**) more than 15 years. Heat maps are split into quadrants based on current/former smokers and pro/anti-inflammatory cytokines. Red bar plots represent proinflammatory cytokines, and blue bar plots represent anti-inflammatory cytokines.

We next examined whether cytokine levels are dysregulated in smokers or e-cig users. We found that current tobacco smokers display significant dysregulation of 18 cytokine genes, including 12 proinflammatory and six anti-inflammatory cytokines, compared to former tobacco smokers (Figure 2A,D,E). Of the proinflammatory cytokines, the following are of particular interest: CCL20, which has been associated with the first severe acute respiratory syndrome coronavirus (SARS-CoV), and IL-1B, CXCL1, CXCL2, and CXCL8, which are associated with COVID-19. CCL20 was highly expressed in healthy Peripheral Blood Mononuclear Cells (PBMCs) infected with SARS-CoV [29]. Given the similarities in the pathophysiology of SARS-CoV and SARS-CoV-2 infection, more research must be done to determine if CCL20 is upregulated in COVID-19 patients as well, thus contributing to a potential cytokine storm. COVID-19 infection of the upper and lower respiratory tract has been shown to release proinflammatory cytokines including IL-1B [30], and is also associated with increased levels of CXCL8, CXCL1, and CXCL2 [31], which are known to play roles in neutrophil recruitment [32,33]. CXCL8 has also been found to be positively correlated with the number of neutrophils recovered in ARDS patients [33]. Such excessive neutrophil recruitment during ARDS is associated with poorer clinical outcomes and greater disease severity [34,35]. Therefore, the upregulation of these cytokines in tobacco smokers may make them more vulnerable to excessive neutrophil migration, a more severe inflammatory response, and leave them more susceptible to cytokine storms. Of the anti-inflammatory cytokines, CXCL17 is upregulated in COVID-19 patients as well [31], but more research must be done to investigate its role in COVID-19 pathogenesis. 

For the e-cig studies, we noticed that there are significant differences between vapers who used nicotine-less and flavor-less e-cig and vapers who used nicotine/flavor-containing e-cig. In nicotine-less and flavor-less e-cig users, we observed only one cytokine, IFNA8, being slightly dysregulated (Figure 2B). However, we observed the dysregulation of six pro-inflammatory cytokines, including CCL5, CCL3, and CXCL3, and two anti-inflammatory cytokines in those who used nicotine/flavor-containing e-cigs (Figure 2C). CCL5 and CCL3 are both chemotactic cytokines that specifically attract T-cells and macrophages to the site of infection [36,37]. CCL5 is also critical to inhibiting macrophages from undergoing apoptosis after the activation of adaptive immunity, potentially leading to prolonged inflammation [37]. CXCL3 is a chemokine released by macrophages and may indicate increased airway inflammation [38]. In total, we found 15 cytokines that were dysregulated when comparing between e-cig smokers, current and former tobacco smokers, and air controls (Table 1). In summary, our data suggest that use of nicotine or flavor-containing e-cigs may lead to greater expression of inflammatory genes. 

### 2.4. Investigation of Inflammasome Activation in E-Cig and Tobacco Users

The upregulation of a significant number of inflammatory cytokines in smokers and nicotine/flavor-containing e-cig users and the association of smoking with IL-1B prompted us to examine inflammasome activation in smokers and e-cig users (Table 2). Inflammasomes are large protein structures primarily located in macrophages that respond to inflammatory signals, namely molecular patterns from pathogens. When activated, inflammasomes cleave pro-IL-1B and pro-IL18 into IL-1B and IL18, allowing them to signal the presence of pathogens to nearby cells and initiate the inflammatory response [39]. The discovery that IL-1B is upregulated by both smoking and COVID-19 suggests the possibility that inflammasomes are activated. 

**Figure 3:**
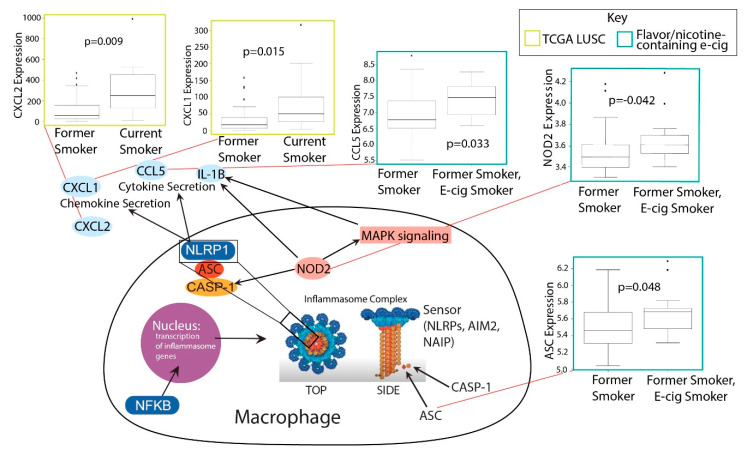
Boxplots of significant correlations of e-cig or tobacco use with upregulation of inflammasome genes (Kruskal–Wallis, *p* < 0.05). A schematic linking the genes upregulated with their function within inflammasome pathways is shown.

We discovered that many key inflammasome genes and regulators besides IL-1B were upregulated in smokers vs. former smokers, including CXCL1 and CXCL2 (Figure 3). These genes are all upregulated in smokers vs. former smokers. Both CXCL1 and CXCL2 promote the activation of inflammasomes [40,41]. In the e-cig studies, we found that flavor-less and nicotine-less e-cig users showed no markers of inflammasome activation (Figure 3). However, users of nicotine/flavor-containing e-cigs also exhibited highly significant upregulation of inflammasome-related genes, including NOD2, CCL5, and ASC (also known as PYCARD) (Figure 3). ASC is one of the three main components of inflammasomes. NOD2 can directly bind to NLRP1 inflammasomes to upregulate IL-1B release [42]. CCL5 expression may be induced by inflammasome activation, as IL-1B has been shown to induce CCL5 expression in lower airway cells [43]. Our data thus suggest that smoking and nicotine or flavor-containing e-cigs, but not nicotine/flavor-less e-cigs, could upregulate inflammasomes and potentially exacerbate COVID-19-induced inflammation. 

## 3. Discussion

The current COVID-19 pandemic intersects with the ongoing epidemic of tobacco smoking and e-cig vaping in the U.S. and across the globe. Tobacco usage has been declining gradually over the years. In total, 42.3% of U.S. adults smoked cigarettes in 1965, but only 13.7% do so in 2018, according to data from the National Health Interview Survey (NHIS). The smoking rate among young adults have also fallen similarly, with 7% of young adults smoking in 2018 [43]. Although these trends are encouraging, it does not mask the fact that millions of Americans are still active smokers. The many detrimental effects tobacco can have on the lungs and other organs have made tobacco usage one of the biggest public health crises the world faces. Accumulating evidence suggests that smokers are more susceptible to bacterial or viral infection and exhibit greater severity of these infections [44,45]. There have also been limited accounts of cigarette smoking increasing severity of COVID-19 infection [45]. The millions of smokers worldwide may be especially vulnerable to COVID-19.

However, more concerning than tobacco usage has been the usage of e-cigs. Although not nearly as popular as tobacco, e-cig usage has been gaining traction at alarming rates. Critically, the usage of e-cigs has been increasing dramatically among teenagers. According to CDC data, more than 1.3 million high school students started using e-cigs in one year alone (2017–2018), which is a 78% increase in usage (11.7% to 20.8%) [46]. From 2011 to 2018, the usage rate among high schoolers increased nearly 14 times (1.5% to 20.8%) [46]. Even among middle schoolers, usage rate increased over eight times from 2011 to 2018 (0.6% to 4.9%) [46]. While the health effects of tobacco are well-known, much less is known about e-cigs. In recent days, starting from late June, the surge in COVID-19 across the U.S. has been significantly attributed to increased incidence amongst the younger segment of the population. Given the prevalence of e-cig vaping in the young population, significant attention must be paid to how e-cig use may be related to COVID-19 incidence and severity. 

Originally marketed as a safer alternative to traditional cigarettes, alarming health risks of e-cigs have been slowly revealed. E-cigs could be cytotoxic to both endothelial and epithelial cells and may decrease immune functions [47]. However, study of e-cigs is complicated by the presence of nicotine and flavorings. Not all e-cigs have nicotine and flavoring, but almost all brands have one or the other. These components can be significantly more cytotoxic than the base ingredients of e-cigs, which is propylene glycol (PG) and vegetable glycerin (VG). While nicotine is known to be cytotoxic, we have previously reported that e-cigs without nicotine can also induce DNA double strand breaks in cells [48].

A possible molecular mechanism for tobacco or e-cigs to increase COVID-19 susceptibility is the upregulation of ACE2. For SARS-CoV-2, host recognition is carried out by the spike protein on the surface of the viral envelope. The spike protein binds the ACE2 receptor protein in human cells. After this, the spike protein can be cleaved by the serine protease TMPRSS2 [49]. SARS-CoV uses a similar mechanism for host recognition, after which, fusion of the viral envelope with membranes in the host cell will allow the virus entry into the cell [50]. This process is vital for the entry of SARS-CoV-2 into human host cells, and therefore plays an integral role in COVID-19 infection and disease progression. Cigarette smoke was found in some studies to increase ACE2 expression in lungs in mammals [17,51,52]. We have previously found that both ACE2 and TMPRSS2 are upregulated in smokers using TCGA samples, although we did not examine e-cig users in that study, and the study focused on the association between ACE2 expression and the androgen pathway [24]. No study has reported on ACE2 expression’s relationship with e-cig use.

In this study, we compared differences in the activation of key molecular pathways related to COVID-19 in tobacco and e-cig users. Specifically, we examined one cohort of e-cig users who only smoked e-cigs without nicotine and without flavorings and another cohort of e-cig users who smoked e-cigs with nicotine and with any flavorings. We found that tobacco use increases ACE2 expression, which corroborates the results of other studies [52]. However, e-cig use in any case did not increase ACE2 or TMPRSS2 expression, which has not been reported in the literature. One recent study exposing mice to nicotine-containing e-cigs for 1 month has found induction of ACE2 after exposure to nicotine-containing e-cigs [53]. In our dataset, humans were also exposed to nicotine-containing e-cigs for at least 1 month, suggesting that the in vivo results may not replicate physiological exposure.

We also found that tobacco use and use of nicotine/flavor-containing e-cigs led to significant cytokine dysregulation and potential inflammasome activation. However, non-flavored and non-nicotine-containing e-cig use does not lead to either. While it has been reported that there is only limited inflammation in users of e-cigs without nicotine and flavors [21], no study has compared gene expression changes of this group of e-cig users to users of nicotine/flavor-containing e-cigs. One study also demonstrated inflammasome activation among e-cig users but did not take into account effects of nicotine and flavors [54].

Several limitations exist in our study that should be addressed in future studies. First, for the tobacco dataset, current smokers were only compared to former smokers, not never smokers. The tobacco dataset is also derived from adjacent tissue of lung squamous cell tumors. While the adjacent normal tissues are not cancerous, immune dysregulation in the cancer tissue may significantly influence the observed inflammatory response. Second, for the e-cig dataset with flavor/nicotine-containing e-cigs, only former smokers, not never smokers, were participants. However, since both the experimental and control group are composed of former smokers, the effect of smoking should not affect the statistical validity of our comparisons. Third, in the e-cig studies, participants were only exposed to e-cigs for at least 1 month, while certain effects of e-cigs may be expected to develop only after chronic exposure. Nonetheless, given that we observed cytokine/inflammasome dysregulations already given the relative short duration of exposure, we may expect chronic exposure to lead to even more dysregulation. 

E-cig manufacturers have proposed that the lack of tobacco in e-cig vapors makes vaping much less deleterious than tobacco smoking. However, chronic vaping is known to correlate to worse outcomes in a variety of diseases. Specifically, in the lungs, vaping has been correlated to toxicity, oxidative stress, and inflammatory response, indicating a positive correlation with general lung damage [55,56]. Additionally, short-term e-cig exposure has been shown to induce changes to small airways in the lung and decreased levels of exhaled nitric oxide (NO) [57]. This latter decrease is associated with changes to inflammation and COPD development, two factors tied to COVID-19-related morbidity [58]. It is still controversial whether vaping definitively contributes to worse disease states for respiratory diseases [59,60]. However, the correlational research between vaping and lung damage indicates that the effects of vaping on COVID-19 infections should be more widely studied before discounting vaping as a significant modulator of severe infections. 

Recent studies into the mechanisms behind SARS-CoV-2 infection have gone beyond interactions with the ACE2 receptor. Development of SARS-CoV-2-specific T cells in individuals not previously infected could indicate another variable controlling infection rate [61]. Epigenetic mechanisms have also been correlated to the ability of the virus to infiltrate the lungs and fuse with epithelial cell membranes. Specifically, syncytium formation by host cells facilitates viral endocytosis but is inhibited via CpG methylation that prevents transcriptional activity of the syncytin-1 protein [62,63]. This mechanism has been studied further in SARS-CoV-2. These studies show that syncytium formation is significantly higher in this strain of coronavirus compared to other coronaviridiae, allowing for a high virulence factor due to higher endocytosis rates [64,65]. Therefore, despite the lack of a correlation between vaping and ACE2 activity, it is possible that vaping may affect COVID-19 infections via an ACE2-independent mechanism. Indeed, vaping has been correlated to a loss of methylation and immune activation [66]. We investigated whether the ACE2-independent mechanism is cytokine dysregulation. Although our findings indicate that e-cigs without flavors and nicotine may only modestly dysregulate cytokine expression, we want to emphasize a cautionary note that most vapers use formulations that contain nicotine and flavorings. Our data indicate that flavors and nicotine may significantly dysregulate cytokine levels and promote inflammation. These data should indicate that vaping may still be a potent risk factor for severe COVID-19 infection depending on the flavor and nicotine content.

In conclusion, our study demonstrated that tobacco and flavored or nicotine-containing e-cig use could both lead to increased inflammatory response, but only tobacco upregulates ACE2. Inflammation and ACE2 upregulation may increase susceptibility to COVID-19. While further experiments and epidemiology data are needed to confirm our results, we believe that our study is a critical early step into evaluating the implications of using tobacco and e-cigs during the COVID-19 pandemic. 

## 4. Materials and Methods 

### 4.1. Datasets of Gene Expression from E-Cig and Tobacco Samples

Bronchial epithelial cell sequencing data from e-cig and non e-cig users were obtained from GSE138326 (*n* = 15 e-cig, 15 non e-cig), provided by Song et al. [21], and GSE112073 (*n* = 15 e-cig, 21 no e-cig), provided by Corbett et al. [22]. In the Song et al. dataset, 30 healthy subjects (21–30 years old) with no prior history of e-cig or tobacco use were randomly assigned to a e-cig vaping group or control group. In the e-cig vaping group, participants used the device twice a day at 20 puffs per session for exactly 4 weeks. E-cig was composed of 50% PG and 50% VG, with no nicotine or flavor. In the Corbett et al. dataset, patients were aged 18–55 years old and were all former cigarette smokers, defined as having been tobacco-abstinent for over 3 months and have smoked at least five cigarettes for 2 years or more at some point of their lives. In total, 15 subjects are former tobacco smokers who have used nicotine-containing e-cig of any brand or flavoring for at least 6 days a week and at least 1 month. A total of 25 subjects are former tobacco smokers who have not used any nicotine-replacement device. Bronchoscopies were performed on all subjects for both studies, and bronchial brushings from the epithelium were profiled by microarray. RNA sequencing data for current and former tobacco smokers were obtained from adjacent normal samples of 49 lung squamous cell carcinoma (LUSC) patients from The Cancer Genome Atlas (TCGA). Subjects were matched for age, gender, and race in both the TCGA and Corbett et al. datasets (Table S1). Song et al. did not provide patient statistics but stated that there were no significant differences in age, gender, and race across cohorts. 

### 4.2. Differential Expression Analysis

Differential expression was assessed between LUSC samples from current smokers and former smokers using the Kruskal–Wallis test (*p* < 0.05). Microarray data from the e-cig datasets were analyzed using both the GEO2R software, which employs the limma (Linear Models for Microarray Analysis) R package, and the Kruskal–Wallis test (*p* < 0.05). Non-smoking control vs. e-cig samples and samples from former smokers vs. former smokers who smoke e-cigs were the comparisons made for the two studies. 

### 4.3. Inference of Immune Cell Infiltration Populations Using Cibersortx

The Cibersortx algorithm (Stanford, California, USA) was be used to deconvolute microarray and RNA-sequencing data to estimate the infiltration levels of 22 immune cell types [25]. The immune cell types examined include naïve B-cells, memory B-cells, plasma cells, CD8 T-cells, CD4 naïve T-cells, CD4 memory resting T-cells, CD4 memory activated T-cells, follicular helper T-cells, regulatory T-cells, gamma-delta T-cells, resting NK cells, activated NK cells, monocytes, M0-M2 macrophages, resting dendritic cells, activated dendritic cells, resting mast cells, activated mast cells, eosinophils, and neutrophils. 

### 4.4. Correlation of Smoking Status/E-Cig Vaping Status to Pathways or Signatures Using GSEA

GSEA (Boston, Massachusetts, USA) was used to determine the correlation to biological pathways or signatures for the same comparisons described in Section 4.1 [67]. A categorical phenotype file was used, with e-cig users, tobacco users, or control as labels. Gene sets were obtained from the Molecular Signatures Database (MSigDB) (Boston, Massachusetts, USA) [68]. Canonical pathways (C2) and immunological signatures (C7) were correlated with vaping/smoking status (*p* < 0.05). 

### 4.5. Selection of Cytokine and Cytokine-Related Genes for Analysis 

Cytokines and cytokine-related included chemokines, chemokine receptors, interleukins, interleukin receptors, interferons, tumor necrosis factors (TNFs), and TGFB family members. 

### 4.6. Selection of Inflammasome-Related Genes for Analysis

Both genes for proteins that constitute the inflammasome complex and genes that regulate the expression of these inflammasome components were included in our analyses. The former includes inflammasome sensor proteins, such as NLRP family proteins, AIM2, and NAIP; adaptor proteins, such as ASC (PYCARD); or effectors, including caspase proteins [39]. The latter includes NF-kB, IKK, MAPK3, and TAK1 [69]. 

## Figures and Tables

**Table 1:** Significantly dysregulated cytokine-related genes.

Gene	Geo2R		Kruskal-Wallis		Cohort
	*p*-Value	logFC	*p*-Value	logFC	
*CCL20*	0.5055	0.1778	0.7851	0.0019	E-cig vs. Former Smokers
	0.3871	−0.1674	0.2907	−0.0177	E-cig vs. None
			0.0076	2.7834	Current vs. Former Tobacco Smokers
*CXCL17*	0.6345	0.0701	0.5315	0.0007	E-cig vs. Former Smokers
	0.5831	−0.0391	0.7968	−0.0010	E-cig vs. None
			0.0353	0.3798	Current vs. Former Tobacco Smokers
*CXCL3*	0.0491	0.1098	0.0650	0.0122	E-cig vs. Former Smokers
	0.9108	0.0071	0.2106	0.0099	E-cig vs. None
			0.0103	1.9380	Current vs. Former Tobacco Smokers
*CXCL8*	0.5096	0.0407	0.4508	0.0093	E-cig vs. Former Smokers
			0.0137	2.1347	Current vs. Former Tobacco Smokers
*IL10RB*	0.1907	−0.1347	0.1051	0.0074	E-cig vs. Former Smokers
	0.0742	−0.0526	0.0439	−0.0021	E-cig vs. None
			0.0481	0.1260	Current vs. Former Tobacco Smokers
*IL10RB-AS1*	0.3466	−0.0597	0.5055	−0.0040	E-cig vs. None
			0.0426	0.3871	Current vs. Former Tobacco Smokers
*IL17D*	0.1372	0.1445	0.1197	0.0152	E-cig vs. Former Smokers
	0.9680	0.0021	0.5639	−0.0017	E-cig vs. None
			0.0182	−0.6035	Current vs. Former Tobacco Smokers
*IL17RD*	0.9196	−0.0084	0.7362	0.0029	E-cig vs. Former Smokers
	0.3403	0.0438	0.4942	0.0004	E-cig vs. None
			0.0017	−1.1050	Current vs. Former Tobacco Smokers
*IL1B*	0.5137	0.1273	0.6649	−0.0052	E-cig vs. Former Smokers
	0.3701	−0.1931	0.7968	−0.0020	E-cig vs. None
			0.0376	0.7497	Current vs. Former Tobacco Smokers
*IL6ST*	0.1507	0.0736	0.4132	−0.0010	E-cig vs. Former Smokers
	0.5503	0.0511	0.8660	0.0016	E-cig vs. None
			0.0353	−0.3333	Current vs. Former Tobacco Smokers
*TGFB2*	0.2557	0.0795	0.9361	−0.0134	E-cig vs. Former Smokers
	0.0231	0.2371	0.1800	0.0022	E-cig vs. None
			0.0040	−1.0251	Current vs. Former Tobacco Smokers
*TGFBR2*	0.2268	0.0852	0.2671	0.0032	E-cig vs. None
			0.0137	−0.3959	Current vs. Former Tobacco Smokers
*TNFAIP8*	0.5235	0.1650	0.1938	−0.0106	E-cig vs. Former Smokers
	0.9072	0.0040	0.6253	0.0003	E-cig vs. None
			0.0453	0.4613	Current vs. Former Tobacco Smokers
*TNFRSF14*	0.2259	−0.1559	0.9361	−0.0009	E-cig vs. Former Smokers
	0.3154	0.0407	0.1289	0.0028	E-cig vs. None
			0.0376	0.3800	Current vs. Former Tobacco Smokers
*TNFSF9*	0.9974	−0.0002	0.4132	0.0010	E-cig vs. Former Smokers
	0.4118	−0.0497	0.3990	−0.0016	E-cig vs. None
			0.0016	1.1718	Current vs. Former Tobacco Smokers

**Table 2:** Significantly dysregulated inflammasome-related gene expression.

Gene	Geo2R		Kruskal-Wallis		Cohort
	*p*-Value	logFC	*p*-Value	logFC	
*CCL5*	0.85415	0.024121	0.273192	0.009959	E-cig vs. None
	0.049094	0.463617	0.032858	0.0421	E-cig vs. Former Smokers
			0.897886	0.077011	Current vs. Former Tobacco Smokers
*CXCL1*	0.622626	0.083064	0.241121	0.016747	E-cig vs. None
	0.274418	0.177215	0.395149	0.00718	E-cig vs. Former Smokers
			0.008846	1.553637	Current vs. Former Tobacco Smokers
*CXCL2*	0.802924	−0.03393	0.429555	0.004948	E-cig vs. None
	0.359789	0.122607	0.413227	0.003556	E-cig vs. Former Smokers
			0.008202	2.113189	Current vs. Former Tobacco Smokers
*NLRP3*	0.995982	0.000195	0.918813	0.002349	E-cig vs. None
	0.183356	0.07004	0.056236	0.013534	E-cig vs. Former Smokers
			0.758085	0.158417	Current vs. Former Tobacco Smokers
*NOD2*	0.317884	0.034773	0.459914	0.001984	E-cig vs. None
	0.207633	0.099462	0.041595	0.014107	E-cig vs. Former Smokers
			0.777691	0.16816	Current vs. Former Tobacco Smokers
*PYCARD*	0.659102	−0.0204	0.721277	−0.00158	E-cig vs. None
	0.048781	0.187701	0.065034	0.017268	E-cig vs. Former Smokers
			0.100456	0.272032	Current vs. Former Tobacco Smokers

## Supplementary Materials

Supplementary materials can be found at https://www.mdpi.com/1422-0067/21/15/5513/s1; Table S1: Patient Characteristics for Each Study.

## Abbreviations

PG	propylene glycol
VG	vegetable glycerin
LUSC	lung squamous cell carcinoma
MSigDB	Molecular Signatures Database
TNF	tumor necrosis factor
limma	Linear Models for Microarray Analysis
Treg	Regulatory T cells
PBMCs	Peripheral Blood Mononuclear Cells
TCGA	The Cancer Genome Atlas

## References

[1] Wu Z., McGoogan J.M. (2020). Characteristics of and important lessons from the coronavirus disease 2019 (COVID-19) outbreak in china: Summary of a report of 72314 cases from the Chinese center for disease control and prevention. JAMA.

[2] Vardavas C.I., Nikitara K. (2020). COVID-19 and smoking: A systematic review of the evidence. Tob. Induc. Dis..

[3] Zhao Q., Meng M., Kumar R., Wu Y., Huang J., Lian N., Deng Y., Lin S. (2020). The impact of COPD and smoking history on the severity of Covid-19: A systemic review and meta-analysis. J. Med. Virol..

[4] Laniado-Laborin R. (2009). Smoking and chronic obstructive pulmonary disease (COPD). Parallel epidemics of the 21 century. Int. J. Environ. Res. Public Health.

[5] Berlin I., Thomas D., Le Faou A.L., Cornuz J. (2020). COVID-19 and smoking. Nicotine Tob. Res..

[6] Farsalinos K., Barbouni A., Niaura R. (2020). Smoking, vaping and hospitalization for COVID-19. Qeios.

[7] Lippi G., Henry B.M. (2020). Active smoking is not associated with severity of coronavirus disease 2019 (COVID-19). Eur. J. Int. Med..

[8] Changeux J.-P., Amoura Z., Rey F., Miyara M. (2020). A nicotinic hypothesis for Covid-19 with preventive and therapeutic implications. C. R. Biol..

[9] Nilsson P., Mallet V. (2020). France limits nicotine sales after coronavirus study. Financial Times.

[10] Bao W., Xu G., Lu J., Snetselaar L.G., Wallace R.B. (2018). Changes in electronic cigarette use among adults in the United States, 2014–2016. JAMA.

[11] McAlinden K.D., Eapen M.S., Lu W., Chia C., Haug G., Sohal S.S. (2020). COVID-19 and vaping: Risk for increased susceptibility to SARS-CoV-2 infection?. Eur. Respir. J..

[12] Reinikovaite V., Rodriguez I.E., Karoor V., Rau A., Trinh B.B., Deleyiannis F.W., Taraseviciene-Stewart L. (2018). The effects of electronic cigarette vapour on the lung: Direct comparison to tobacco smoke. Eur. Respir. J..

[13] Muthumalage T., Lamb T., Friedman M.R., Rahman I. (2019). E-cigarette flavored pods induce inflammation, epithelial barrier dysfunction, and DNA damage in lung epithelial cells and monocytes. Sci. Rep..

[14] Mehta P., McAuley D.F., Brown M., Sanchez E., Tattersall R.S., Manson J.J., Hlh Across Speciality Collaboration U.K. (2020). COVID-19: Consider cytokine storm syndromes and immunosuppression. Lancet.

[15] Vaninov N. (2020). In the eye of the COVID-19 cytokine storm. Nat. Rev. Immunol..

[16] Lee J., Taneja V., Vassallo R. (2012). Cigarette smoking and inflammation: Cellular and molecular mechanisms. J. Dent. Res..

[17] Smith J.C., Sheltzer J.M. (2020). Cigarette smoke triggers the expansion of a subpopulation of respiratory epithelial cells that express the SARS-CoV-2 receptor ACE2. bioRxiv.

[18] Scott A., Lugg S.T., Aldridge K., Lewis K.E., Bowden A., Mahida R.Y., Grudzinska F.S., Dosanjh D., Parekh D., Foronjy R. (2018). Pro-inflammatory effects of e-cigarette vapour condensate on human alveolar macrophages. Thorax.

[19] Reidel B., Radicioni G., Clapp P.W., Ford A.A., Abdelwahab S., Rebuli M.E., Haridass P., Alexis N.E., Jaspers I., Kesimer M. (2018). E-cigarette use causes a unique innate immune response in the lung, involving increased neutrophilic activation and altered mucin secretion. Am. J. Respir. Crit. Care Med..

[20] Guo H., Callaway J.B., Ting J.P. (2015). Inflammasomes: Mechanism of action, role in disease, and therapeutics. Nat. Med..

[21] Song M.A., Reisinger S.A., Freudenheim J.L., Brasky T.M., Mathe E.A., McElroy J.P., Nickerson Q.A., Weng D.Y., Wewers M.D., Shields P.G. (2020). Effects of electronic cigarette constituents on the human lung: A pilot clinical trial. Cancer Prev. Res. Phila.

[22] Corbett S.E., Nitzberg M., Moses E., Kleerup E., Wang T., Perdomo C., Perdomo C., Liu G., Xiao X., Liu H. (2019). Gene expression alterations in the bronchial epithelium of E-Cigarette users. Chest.

[23] Lukassen S., Chua R.L., Trefzer T., Kahn N.C., Schneider M.A., Muley T., Winter H., Meister M., Veith C., Boots A.W. (2020). SARS-CoV-2 receptor ACE2 and TMPRSS2 are primarily expressed in bronchial transient secretory cells. EMBO J..

[24] Chakladar J., Shende N., Li W.T., Rajasekaran M., Chang E.Y., Ongkeko W.M. (2020). Smoking-Mediated Upregulation of the Androgen Pathway Leads to Increased SARS-CoV-2 Susceptibility. Int. J. Mol. Sci..

[25] Chen B., Khodadoust M.S., Liu C.L., Newman A.M., Alizadeh A.A. (2018). Profiling tumor infiltrating immune cells with CIBERSORT. Methods Mol. Biol..

[26] Dutta A., Miaw S.C., Yu J.S., Chen T.C., Lin C.Y., Lin Y.C., Chang C.S., He Y.C., Chuang S.H., Yen M.I. (2013). Altered T-bet dominance in IFN-gamma-decoupled CD4+ T cells with attenuated cytokine storm and preserved memory in influenza. J. Immunol..

[27] Gogishvili T., Langenhorst D., Luhder F., Elias F., Elflein K., Dennehy K.M., Gold R., Hunig T. (2009). Rapid regulatory T-cell response prevents cytokine storm in CD28 superagonist treated mice. PLoS ONE.

[28] Brandsma C.A., Hylkema M.N., Geerlings M., van Geffen W.H., Postma D.S., Timens W., Kerstjens H.A. (2009). Increased levels of (class switched) memory B cells in peripheral blood of current smokers. Respir. Res..

[29] Ng L.F., Hibberd M.L., Ooi E.E., Tang K.F., Neo S.Y., Tan J., Murthy K.R., Vega V.B., Chia J.M., Liu E.T. (2004). A human in vitro model system for investigating genome-wide host responses to SARS coronavirus infection. BMC Infect. Dis..

[30] Conti P., Ronconi G., Caraffa A., Gallenga C.E., Ross R., Frydas I., Kritas S.K. (2020). Induction of pro-inflammatory cytokines (IL-1 and IL-6) and lung inflammation by Coronavirus-19 (COVI-19 or SARS-CoV-2): Anti-inflammatory strategies. J. Biol. Regul. Homeost. Agents.

[31] Zhou Z., Ren L., Zhang L., Zhong J., Xiao Y., Jia Z., Guo L., Yang J., Wang C., Jiang S. (2020). Heightened innate immune responses in the respiratory Tract of COVID-19 patients. Cell Host Microbe.

[32] Donnelly S.C., Strieter R.M., Kunkel S.L., Walz A., Robertson C.R., Carter D.C., Grant I.S., Pollok A.J., Haslett C. (1993). Interleukin-8 and development of adult respiratory distress syndrome in at-risk patient groups. Lancet.

[33] Miller E.J., Cohen A.B., Nagao S., Griffith D., Maunder R.J., Martin T.R., Weiner-Kronish J.P., Sticherling M., Christophers E., Matthay M.A. (1992). Elevated levels of NAP-1/interleukin-8 are present in the airspaces of patients with the adult respiratory distress syndrome and are associated with increased mortality. Am. Rev. Respir. Dis..

[34] Groeneveld A.B., Raijmakers P.G., Hack C.E., Thijs L.G. (1995). Interleukin 8-related neutrophil elastase and the severity of the adult respiratory distress syndrome. Cytokine.

[35] Miller E.J., Cohen A.B., Matthay M.A. (1996). Increased interleukin-8 concentrations in the pulmonary edema fluid of patients with acute respiratory distress syndrome from sepsis. Crit. Care Med..

[36] Trifilo M.J., Bergmann C.C., Kuziel W.A., Lane T.E. (2003). CC chemokine ligand 3 (CCL3) regulates CD8(+)-T-cell effector function and migration following viral infection. J. Virol..

[37] Tyner J.W., Uchida O., Kajiwara N., Kim E.Y., Patel A.C., O’Sullivan M.P., Walter M.J., Schwendener R.A., Cook D.N., Danoff T.M. (2005). CCL5-CCR5 interaction provides antiapoptotic signals for macrophage survival during viral infection. Nat. Med..

[38] Tekamp-Olson P., Gallegos C., Bauer D., McClain J., Sherry B., Fabre M., van Deventer S., Cerami A. (1990). Cloning and characterization of cDNAs for murine macrophage inflammatory protein 2 and its human homologues. J. Exp. Med..

[39] Lu A., Wu H. (2015). Structural mechanisms of inflammasome assembly. FEBS J..

[40] Boro M., Balaji K.N. (2017). CXCL1 and CXCL2 regulate NLRP3 inflammasome activation via G-Protein-Coupled receptor CXCR2. J. Immunol..

[41] Rajamaki K., Mayranpaa M.I., Risco A., Tuimala J., Nurmi K., Cuenda A., Eklund K.K., Oorni K., Kovanen P.T. (2016). p38delta MAPK: A novel regulator of NLRP3 inflammasome activation with increased expression in coronary atherogenesis. Arter. Thromb. Vasc. Biol..

[42] Hsu L.C., Ali S.R., McGillivray S., Tseng P.H., Mariathasan S., Humke E.W., Eckmann L., Powell J.J., Nizet V., Dixit V.M. (2008). A NOD2-NALP1 complex mediates caspase-1-dependent IL-1beta secretion in response to Bacillus anthracis infection and muramyl dipeptide. Proc. Natl. Acad. Sci. USA.

[43] Williford J., Zablotsky B., Zelaya C. (2019). QuickStats: Percentage of adults aged 18–24 years who currently smoke cigarettes* or who currently use electronic cigarettes, (dagger) by Year—National Health Interview Survey, United States, 2014–2018 (section sign). Morb. Mortal. Wkly. Rep..

[44] Bagaitkar J., Demuth D.R., Scott D.A. (2008). Tobacco use increases susceptibility to bacterial infection. Tob. Induc. Dis..

[45] Van Zyl-Smit R.N., Richards G., Leone F.T. (2020). Tobacco smoking and COVID-19 infection. Lancet Respir. Med..

[46] Cullen K.A., Ambrose B.K., Gentzke A.S., Apelberg B.J., Jamal A., King B.A. (2018). Notes from the Field: Use of electronic cigarettes and any tobacco product among middle and high school students—United States, 2011–2018. Morb. Mortal. Wkly. Rep..

[47] Chaffee B.W. (2019). Electronic cigarettes: Trends, health effects and advising patients amid uncertainty. J. Calif Dent. Assoc..

[48] Yu V., Rahimy M., Korrapati A., Xuan Y., Zou A.E., Krishnan A.R., Tsui T., Aguilera J.A., Advani S., Crotty Alexander L.E. (2016). Electronic cigarettes induce DNA strand breaks and cell death independently of nicotine in cell lines. Oral Oncol..

[49] Hoffmann M., Kleine-Weber H., Schroeder S., Krüger N., Herrler T., Erichsen S., Schiergens T.S., Herrler G., Wu N.-H., Nitsche A. (2020). SARS-CoV-2 cell entry depends on ACE2 and TMPRSS2 and is blocked by a clinically proven protease inhibitor. Cell.

[50] Glowacka I., Bertram S., Müller M.A., Allen P., Soilleux E., Pfefferle S., Steffen I., Tsegaye T.S., He Y., Gnirss K. (2011). Evidence that TMPRSS2 activates the severe acute respiratory syndrome coronavirus spike protein for membrane fusion and reduces viral control by the humoral immune response. J. Virol..

[51] Leung J.M., Yang C.X., Tam A., Shaipanich T., Hackett T.L., Singhera G.K., Dorscheid D.R., Sin D.D. (2020). ACE-2 Expression in the small airway epithelia of smokers and COPD patients: Implications for COVID-19. Eur. Respir. J..

[52] Cai G., Bosse Y., Xiao F., Kheradmand F., Amos C.I. (2020). Tobacco smoking increases the lung gene Expression of ACE2, the receptor of SARS-CoV-2. Am. J. Respir Crit Care Med..

[53] Wang Q., Sundar I.K., Li D., Lucas J.H., Muthumalage T., McDonough S.R., Rahman I. (2020). E-cigarette-induced pulmonary inflammation and dysregulated repair are mediated by nAChR alpha7 receptor: Role of nAChR alpha7 in SARS-CoV-2 Covid-19 ACE2 receptor regulation. Respir. Res..

[54] Tsai M., Song M.A., McAndrew C., Brasky T.M., Freudenheim J.L., Mathe E., McElroy J., Reisinger S.A., Shields P.G., Wewers M.D. (2019). Electronic versus combustible cigarette effects on inflammasome component release into human lung. Am. J. Respir. Crit. Care Med..

[55] Lerner C.A., Sundar I.K., Yao H., Gerloff J., Ossip D.J., McIntosh S., Rahman I. (2015). Vapors produced by electronic cigarettes and e-juices with flavorings induce toxicity, oxidative stress, and inflammatory response in lung epithelial cells and in mouse lung. PLoS ONE.

[56] Chaumont M., van de Borne P., Bernard A., Van Muylem A., Deprez G., Ullmo J., Debbas N. (2019). Fourth generation e-cigarette vaping induces transient lung inflammation and gas exchange disturbances: Results from two randomized clinical trials. Am. J. Physiol. Lung Cell. Mol. Physiol..

[57] Rowell T.R., Tarran R. (2015). Will chronic e-cigarette use cause lung disease?. Am. J. Physiol. Lung Cell. Mol. Physiol..

[58] Zhou M., Liu Y., Duan Y. (2012). Breath biomarkers in diagnosis of pulmonary diseases. Clin. Chim. Acta.

[59] Pisinger C., Døssing M. (2014). A systematic review of health effects of electronic cigarettes. Prev. Med..

[60] Rom O., Pecorelli A., Valacchi G., Reznick A.Z. (2015). Are E-cigarettes a safe and good alternative to cigarette smoking?. Ann. N. Y. Acad. Sci..

[61] Le Bert N., Tan A.T., Kunasegaran K., Tham C.Y., Hafezi M., Chia A., Chia W.N. (2020). SARS-CoV-2-specific T cell immunity in cases of COVID-19 and SARS, and uninfected controls. Nature.

[62] Levet S., Charvet B., Bertin A., Deschaumes A., Perron H., Hober D. (2019). Human endogenous retroviruses and Type 1 Diabetes. Curr. Diab. Rep..

[63] Matousková M., Blazková J., Pajer P., Pavlícek A., Hejnar J. (2006). CpG methylation suppresses transcriptional activity of human syncytin-1 in non-placental tissues. Exp. Cell Res..

[64] Xia S., Liu M., Wang C., Xu W., Lan Q., Feng S., Qin C. (2020). Inhibition of SARS-CoV-2 (previously 2019-nCoV) infection by a highly potent pan-coronavirus fusion inhibitor targeting its spike protein that harbors a high capacity to mediate membrane fusion. Cell Res..

[65] Matsuyama S., Nao N., Shirato K., Kawase M., Saito S., Takayama I., Sakata M. (2020). Enhanced isolation of SARS-CoV-2 by TMPRSS2-expressing cells. Proc. Natl. Acad. Sci. USA.

[66] Caliri A.W., Caceres A., Tommasi S., Besaratinia A. (2020). Hypomethylation of LINE-1 repeat elements and global loss of DNA hydroxymethylation in vapers and smokers. Epigenetics.

[67] Subramanian A., Tamayo P., Mootha V.K., Mukherjee S., Ebert B.L., Gillette M.A., Paulovich A., Pomeroy S.L., Golub T.R., Lander E.S. (2005). Gene set enrichment analysis: A knowledge-based approach for interpreting genome-wide expression profiles. Proc. Natl. Acad. Sci. USA.

[68] Liberzon A., Subramanian A., Pinchback R., Thorvaldsdottir H., Tamayo P., Mesirov J.P. (2011). Molecular signatures database (MSigDB) 3.0. Bioinformatics.

[69] Song N., Li T. (2018). Regulation of NLRP3 Inflammasome by Phosphorylation. Front. Immunol..

